# Cut‐Off Points for Low Relative 30‐s Sit‐to‐Stand Power and Their Associations With Adverse Health Conditions

**DOI:** 10.1002/jcsm.13676

**Published:** 2025-01-10

**Authors:** Mikel Garcia‐Aguirre, Ivan Baltasar‐Fernandez, Julian Alcazar, Jose Losa‐Reyna, Ana Alfaro‐Acha, Ignacio Ara, Leocadio Rodriguez‐Mañas, Luis M. Alegre, Francisco J. Garcia‐Garcia

**Affiliations:** ^1^ GENUD Toledo Research Group, Faculty of Sport Sciences University of Castilla‐La Mancha Toledo Spain; ^2^ Centro de Investigación Biomédica en Red Fragilidad y Envejecimiento Saludable (CIBERFES) Instituto de Salud Carlos III Madrid Spain; ^3^ Grupo Mixto de Fragilidad y Envejecimiento Exitoso UCLM‐SESCAM Universidad de Castilla‐La Mancha‐Servicio de Salud de Castilla‐La Mancha, IDISCAM Toledo Spain; ^4^ Faculty of Health Sciences University of Castilla‐La Mancha Talavera de la Reina Spain; ^5^ Valoración del Rendimiento Deportivo, Actividad Física y Salud y Lesiones Deportivas (REDAFLED) Universidad de Valladolid (Campus Duques de Soria) Valladolid Spain; ^6^ Geriatrics Department Hospital Universitario de Toledo Toledo Spain; ^7^ Geriatrics Department Getafe University Hospital Getafe Spain

**Keywords:** ageing, disability, frailty, functional decline, healthy ageing, muscle power

## Abstract

**Background:**

Despite muscle power derived from the 5‐rep sit‐to‐stand (STS) test having been demonstrated to be a valuable biomarker in older individuals, there is limited information regarding muscle power derived from the 30‐s STS test, a widely used test in the clinical setting. This study aimed (i) to compare relative 30‐s STS power values between older men and women, (ii) to identify cut‐off points for low relative 30‐s STS power, (iii) to compare the prevalence of low relative STS power between sexes and (iv) to evaluate the association of low relative 30‐s STS power with adverse conditions in older people.

**Methods:**

A total of 1475 community‐dwelling older adults (65–98 years; 45% men) from the Toledo Study for Healthy Aging were included. Relative STS power was assessed using the 30‐s STS test and the Alcazar's equation. Adverse health conditions considered encompassed frailty, depression, disability in basic (BADL) and instrumental activities of daily living (IADL), cognitive impairment and low habitual gait speed (HGS).

**Results:**

Relative STS power decreased linearly at an average rate of 1.0% year^−1^ in men and 1.5% year^−1^ in women. The cut‐off points for low relative STS power were 2.53 and 2.01 W·kg^−1^ for men and women, respectively. The prevalence of low relative STS power was significantly lower in older men compared with older women (43.5% vs. 50.0%, respectively; *p* = 0.005). In men, low relative STS power was associated with frailty (OR [95% CI] = 4.4 [2.4–8.0]), cognitive impairment (OR [95% CI] = 1.7 [1.0–2.7]), disability in BADL (OR [95% CI] = 4.5 [1.5–13.8]) and low HGS (OR [95% CI] = 3.4 [1.9–5.9]). In women, low relative STS power was associated with frailty (OR [95% CI] = 5.2 [3.5–7.7]), disability in BADL (OR [95% CI] = 4.3 [1.8–9.9]) and IADL (OR [95% CI] = 3.1 [2.2–4.3]) and low HGS (OR [95% CI] = 6.1 [2.8–13.1]). No associations were found between low relative STS power and disability in IADL or depression in men, nor between low relative STS power and cognitive impairment or depression in women.

**Conclusion:**

Relative STS power decreased with increasing age in both men and women. The provided sex‐specific cut‐off points for low relative STS power using the 30‐s STS test adequately identified older people with frailty and were associated with an increased risk of experiencing adverse conditions.

## 1 Introduction

Quality of life and medical advancements during the last century have led to a significant increase in human life expectancy [1, 2]. This improvement has been observed on a global scale, notably impacting the ageing population, particularly in Europe and North America [S1, 3], and leading to a predicted 1.5 billion people over 65 years of age by 2050 [2]. Ageing is a complex physiological process caused by numerous biological and genetic alterations associated with functional decline and increased risk of syndromes or diseases [4]. Frailty is defined as a biological syndrome, a recognized state of increased vulnerability caused by the loss of functional reserves across several physiological systems [5]. Several studies have highlighted the relevance of its assessment, proposing various scales [5, 6, S2, S3] and demonstrating a strong association between frailty and multiple diseases and adverse conditions, including cognitive impairment, disability, depression, musculoskeletal disorders and mortality [S4].

The deterioration of the musculoskeletal system is closely linked to ageing and is considered a critical factor in the development of frailty, disability and mortality [7, 8]. In this sense, sarcopenia is an age‐related syndrome characterized by the loss of both skeletal muscle mass and strength [7]. This syndrome is estimated to impact approximately 10% of healthy older adults (> 60 years); however, in older individuals with frailty, the prevalence of sarcopenia rises to 33% [S5]. Nevertheless, relative muscle power (i.e., muscle power normalized to body mass) has been recently demonstrated to decline earlier and at a faster rate than both muscle strength and muscle mass [9] and to be a stronger predictor of adverse conditions than sarcopenia [10].

Relative muscle power can be assessed in a straightforward, rapid, inexpensive and effective manner through the sit‐to‐stand (STS) test and the implementation of the equation developed by Alcazar et al. [11]. The Alcazar's equation has been validated against various gold standard instruments for muscle power assessment and in diverse groups of older individuals, from well‐functioning to mobility‐limited [12], proving to be the most indicative equation for adverse conditions among all equations found in the literature [13]. In this context, several recent investigations have provided relative STS power cut‐off points for older individuals with mobility limitations [14], frailty [15] or falls [16] using the 5‐repetition version of the STS test. Nevertheless, the 5‐repetition STS version may present a limitation in older individuals with mobility limitations or frailty because 26% of older women and 13% of older men are unable to perform five consecutive STS repetitions [17]. In this context, the 30‐s version of the STS test may be superior, as it assesses the times a person rises from a chair within a 30‐s timeframe, including individuals who may not reach the threshold of five repetitions [S6]. However, despite the strong association between low relative STS power and various adverse conditions and the widespread use of the 30‐s STS test among older adults, there is no previous research reporting cut‐off points for low relative STS power using the 30‐s STS version and the potential relationship between low relative STS power and adverse conditions.

Therefore, the main aims of this study were (1) to compare relative 30‐s STS power values between men and women across the older adult lifespan, (2) to identify cut‐off points for low relative 30‐s STS power based on their ability to discriminate between frail and non‐frail older adults, (3) to compare the prevalence of low relative 30‐s STS power between sexes and (4) to evaluate the associations of low relative 30‐s STS power with frailty, depression, disability in basic (BADL) and instrumental activities of daily living (IADL), cognitive impairment and low habitual gait speed (HGS) in older men and women.

## 2 Materials and Methods

### 2.1 Study Design and Participants

The Toledo Study for Healthy Aging is a population‐based prospective cohort study designed to evaluate cognitive, functional and frailty parameters in community‐dwelling older adults (> 65 years) of the province of Toledo, Spain [18]. The present study included cross‐sectional data collected from participants between 2006 and 2009. Participants were randomly selected and recruited by two‐stage random sampling. In total, 1475 participants (65–98 years; 806 women and 669 men) completed all the required testing and were included in the current analyses. The participants signed an informed consent, and the Clinical Research Ethics Committee of the Toledo Hospital (Spain) approved the study protocol (reference number 22). All the study procedures were conducted according to the Helsinki Declaration.

### 2.2 Anthropometrics

Height was measured with a portable stadiometer with a precision of 1 mm (Medizintechnikseit 1890; KaWe, Asperg, Germany), while body mass was measured with a scale device with a precision of 0.1 kg (Seca 711, Hamburg, Germany). Participants were required to wear light clothing and remove their footwear beforehand. Then, body mass index was calculated as body mass divided by squared height (kg·m^−2^).

### 2.3 Relative STS Power

STS power was evaluated using the 30‐s STS test. After the cue ‘ready, set, go!’, the participants were instructed to perform the maximum number of STS repetitions within 30 s on a standardized 0.43 m chair without armrests. Participants had to perform the test with arms crossed over the chest, and the STS repetitions were considered valid if the participant stood up completely (full knee and hip extension) and at least touched the chair with the buttocks upon sitting. The maximum number of repetitions performed in the 30‐s STS test was recorded, and subsequently, the Alcazar's equation was used to calculate 30‐s absolute STS power [19]:

$$\begin{array}{c} 30-s\;\text{absolute}\;\mathrm{STS}\;\text{power}\;\left(W\right)=\\ \left(\text{Body mass}\;\left(\mathrm{kg}\right)\cdot 0.9\cdot g\right)\cdot \frac{\left(\left[\text{Body height}\;\left(m\right)\cdot 0.5\right]-\text{Chair height}\;\left(m\right)\right)}{\left(\frac{30\;s}{\text{number of}\;\mathrm{STS}\;\text{repetitions}}\right)\cdot 0.5} \end{array}$$

Then, 30‐s relative STS power was computed by dividing absolute STS power by body mass (W·kg^−1^).

### 2.4 Frailty

Frailty was evaluated with two different scales: (1) the frailty trait scale‐short form (FTS5) [9] and (2) the Fried frailty phenotype (FP) [5]. The FTS5 is a scale that assesses five different items (body mass index, physical activity, handgrip strength, static balance and HGS), providing a final score ranging from 0 to 50 points. Frailty is considered in participants with scores > 25 points [6]. The FP includes five frailty criteria (self‐reported exhaustion, weakness, unintentional weight loss, slowness and low physical activity) and classifies the participants as robust (0 criteria), pre‐frail (1–2 criteria) or frail (3–5 criteria) [5].

### 2.5 Disability in Activities of Daily Living, Cognitive Impairment, Depression, Low HGS and Comorbidities

BADL and IADL were evaluated by the Katz index of independence in activities of daily living [20] and the Lawton and Brody scale [21], respectively. The Katz index is a questionnaire that assesses the functional status in six BADL: bathing, continence, feeding, toilet use, dressing and chair/bed transfer. The total score ranges from 0 (dependence in all BADL) to 6 (independence in all BADL). The Lawton and Brody scale assesses a person's ability to perform eight different IADL: telephone usage, laundry, shopping, transportation, food preparation, medication usage, housekeeping and finances. The total score ranges from 0 (dependence in all IADL) to 8 (independence in all IADL). Older adults with disability in BADL and IADL were considered when the Katz index was ≤ 5 points and when the Lawton and Brody scale was ≤ 7 points, respectively.

Cognitive performance was assessed with the mini‐mental state examination (MMSE) [22]. The questionnaire required participants to respond to 11 items assessing six distinct cognitive domains (orientation, repetition, verbal recall, attention and calculation, language and visual construction). The maximum achievable score is 30 points. Given that the majority of participants in our sample had not completed primary education, a cut‐off point of < 20 was selected to indicate cognitive impairment [22].

Depression was assessed through the Geriatric Depression Scale (GDS) [23]. This scale has been extensively used in older adults and is based on a 15‐question dichotomous questionnaire. Scores range from 0 to 15, with scores ≥ 5 points indicating depression [S7].

HGS was evaluated with the 3‐m HGS [24]. Participants were asked to walk at their HGS along a 3‐m distance. The time needed to complete the 3‐m distance was recorded using a stopwatch to the nearest 0.01 s. Low HGS was considered when HGS < 0.8 m·s^−1^ [24].

Comorbidities were assessed using the Charlson comorbidity index [25], a validated scale designed to quantify the number and severity of comorbidities and their impact on patient survival. A higher score denotes an increased burden of comorbidities and, consequently, an increased risk of mortality.

### 2.6 Statistical Analysis

Descriptive data are shown as mean ± standard deviation (SD) for continuous variables, whereas categorical variables are expressed as frequencies (*n*) with corresponding percentage (%) values. The normality distribution was examined using the Kolmogorov–Smirnov test, while the homoscedasticity was evaluated using Levene's test. Baseline differences between men and women were analysed using Student's *t*‐test for independent samples. A two‐way ANOVA with two fixed effects (sex and quinquennial age groups) was used for assessing differences in relative STS power between men and women across the different quinquennial age groups (65–69, 70–74, 75–79, 80–84 and ≥ 85 years). Pairwise comparisons were conducted using Bonferroni's post hoc tests. Segmented stepwise linear regression analyses were used to examine the relationship between age and relative STS power in men and women. An iterative approach was used to detect the potential age points at which a change in slope occurred (70, 75, 80, 85, 90 years old) across various age intervals (65–75, 65–80, 70–90, 75–100, 80–100 years old). Only age points exhibiting a significant change in the slope were incorporated into the final regression model. The relationship between 30‐s relative STS power and frailty (FTS5) was examined through simple regression analysis. Receiver operator characteristic (ROC) curves were then used to determine the optimal cut‐off points for low 30‐s relative STS power among older adult who were able to stand up from the chair according to their ability to discriminate between frail and non‐frail older adults as defined by the FTS5. Area under the curve (AUC) values were reported, and optimal cut‐off points were obtained based on the best trade‐off (product) between sensitivity and specificity. Then, differences in the prevalence of low 30‐s relative STS power between men and women across various age groups were assessed using the χ^2^ test. Binary logistic regression models adjusted for age and comorbidities were used in both men and women to analyse the association between low 30‐s relative STS power with adverse conditions. All statistical analyses were performed using SPSS v21 (SPSS Inc., Chicago, IL), and the significance level was set at α = 0.05.

## 3 Results

The baseline characteristics of the participants are presented in Table 1. Older men exhibited higher body mass, height, disability in IADL, cognitive capacity, HGS and relative 30‐s STS power values but lower body mass index, frailty trait scale 5, BADL activities of daily living, depression and Charlson index values compared with older women (all *p* < 0.05). There were no sex differences in terms of age and FP (all *p* > 0.05).

**TABLE 1:** Baseline characteristics of study participants.

	All participants (*N* = 1475)	Men (*n* = 669)	Women (*n* = 806)
	Mean ± SD	Mean ± SD	Mean ± SD
Age (years)	74.3 ± 5.5	74.4 ± 5.4	74.3 ± 5.5
Weight (kg)	72.5 ± 12.4	76.4 ± 11.8	69.2 ± 12.0*
Height (m)	1.58 ± 0.1	1.64 ± 0.1	1.52 ± 0.1*
Body mass index (kg·m ^2^ )	29.1 ± 4.5	28.2 ± 3.9	29.8 ± 4.9*
FTS5 (points)	19.3 ± 7.2	17.2 ± 6.8	20.9 ± 7.1*
Frailty FTS5, *n* (%)	303 (21.4%)	76 (12.0%)	227 (29.1%)*
FP (*n* of criteria)	0.69 ± 0.88	0.69 ± 0.92	0.7 ± 0.9
Frailty FP, *n* (%)	68 (4.8%)	27 (4.0%)	41 (5.3%)
Katz index (points)	5.92 ± 0.43	5.94 ± 0.36	5.89 ± 0.48*
Disability in BADL, *n* (%)	79 (5.4%)	22 (3.3%)	57 (7.2%)*
L&B scale (points)	6.4 ± 1.8	5.8 ± 1.8	7.1 ± 1.5*
Disability in IADL, *n* (%)	774 (56.8%)	451 (78.3%)	324 (41.0%)*
GDS (points)	2.3 ± 2.6	1.6 ± 1.8	2.8 ± 3.0*
Depression, *n* (%)	202 (15.4%)	52 (8.8%)	150 (20.8%)*
MMSE (points)	24.0 ± 4.9	24.6 ± 4.6	23.6 ± 5.1*
Cognitive impairment, *n* (%)	246 (19.1%)	90 (15.5%)	156 (22.1%)*
Habitual gait speed (m·s^−1^)	0.56 ± 0.20	0.64 ± 0.22	0.59 ± 0.21*
Low HGS, *n* (%)	1287 (88.0%)	563 (84.9%)	724 (90.6%)*
Relative STS power (W·kg ^−1^ )	2.38 ± 0.9	2.75 ± 0.94	2.07 ± 0.70*
Charlson index (points)	1.7 ± 1.8	1.6 ± 1.8	1.8 ± 1.7*

*Note:* A total of 1414 (633 men and 781 women) FTS5; 1423 (646 men and 777 women) FP; 1462 (666 men and 796 women) BADL; 1363 (576 men and 787 women) IADL; 1311 (590 men and 721 women) GDS; 1285 (580 men and 705 women) MMSE; 1462 (663 men and 799 women) HGS participants were evaluated for each variable.

Abbreviations: BADL, basic activities of daily living; FP, frailty phenotype; FTS5, Frailty Trait Scale Short Form; GDS, Geriatric Depression Scale; HGS, habitual gait speed; IADL, instrumental activities of daily living; L&B scale, Lawton and Brody Scale; MMSE, mini‐mental state examination; STS, sit‐to‐stand.

* denotes significant differences compared with men (*p* < 0.05).

### 3.1 Differences in Relative 30‐s STS Power Between Men and Women by Age Group

The main differences in 30‐s relative STS power between men and women across age groups are presented in Table 2. Older men exhibited higher 30‐s relative STS power values than women across all age groups (*p* < 0.01). In addition, all age groups aged over 70 years showed significant differences with the 65‐ to 69‐year‐old group. The group of older men aged > 85 years showed −0.63 W·kg^−1^ (−21%) less than the 65‐ to 69‐year‐old group, while older women aged > 85 years exhibited −0.70 W·kg^−1^ (−30%) less than older women aged 65–69 years. No significant differences were observed among the other subsequent age groups, in both men and women (*p* > 0.05).

**TABLE 2:** Comparison of relative STS power values between men and women by age group.

	Men				Women			
	Relative STS power (W·kg ^−1^ )				Relative STS power (W·kg ^−1^ )			
Age group	*N*	Mean ± SD	Difference ^a^		*N*	Mean ± SD	Difference ^a^	
			W·kg^−1^	%			W·kg^−1^	%
65–69 years	(141)	3.06 ± 0.98			(175)	2.33 ± 0.74^#^		
70–74 years	(216)	2.84 ± 0.96*	**−0.22**	**−7.2**	(263)	2.16 ± 0.69*, ^#^	**−0.17**	**−7.3**
75–79 years	(201)	2.63 ± 0.92*	**−0.21**	**−7.4**	(234)	1.98 ± 0.66*, ^#^	**−0.18**	**−8.3**
80–84 years	(84)	2.42 ± 0.72*	**−0.21**	**−8.0**	(97)	1.74 ± 0.53*, ^#^	**−0.24**	**−12.1**
≥ 85 years	(27)	2.43 ± 0.77*	+0.01	+0.4	(37)	1.63 ± 0.46*, ^#^	**−0.11**	**−6.3**

*Note:* Bold values indicate significant changes relative to the preceding quinquennial age group within the same sex (*p* < 0.05).

^a^ Absolute (W·kg^−1^) and percentual (%) difference with respect to the previous quinquennial age group.

* Significantly different compared with the 65–69 years old group within the same sex (*p* < 0.05).

# Significantly different compared with men within the same age group (*p* < 0.01).

**FIGURE 1:**
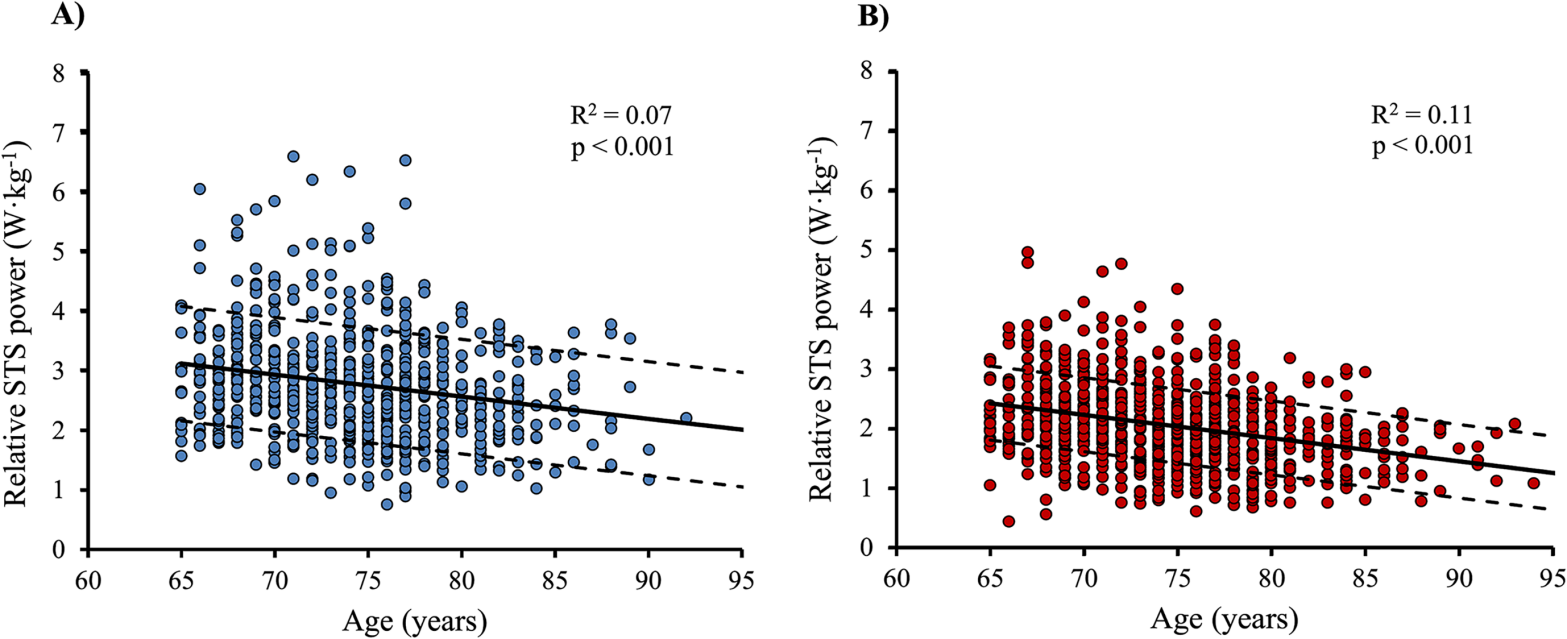
Association between relative STS power and age in older men (A) and women (B).

Both older men (β = −0.037; *R* ^2^ = 0.07; *p* < 0.001) and women (β = −0.039; *R* ^2^ = 0.11; *p* < 0.001) showed a linear and negative association between 30‐s relative STS power and age (Figure 1A,B, respectively). The stepwise regression analysis did not show any significant change in the slope (all *p* > 0.05).

### 3.2 Low Relative 30‐s STS Power Cut‐Off Points

**FIGURE 2:**
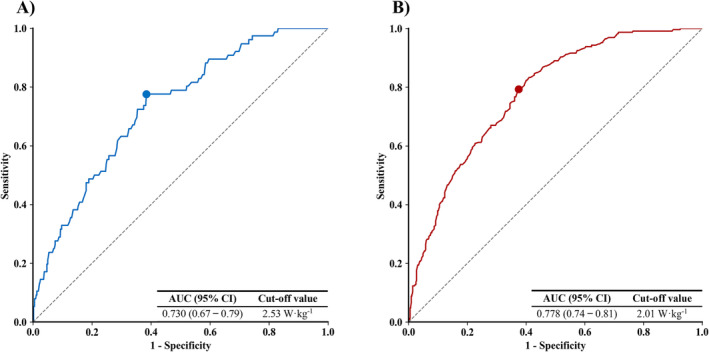
Receiver operator characteristic curve plots compare how relative STS power allows the identification of older men (A) and women (B) with frailty (FTS5 ≥ 25). 95% CI, 95% confidence interval; AUC, area under the curve; FTS5, Frailty Trait Scale Short Form.

A significant and moderate negative correlation was observed between 30‐s relative STS power and the FTS5 in both men (β = −3.28; *R* ^2^ = 0.211; *p* < 0.001) and women (β = −5.61; *R* ^2^ = 0.310; *p* < 0.001). The optimal cut‐off point of low 30‐s relative STS power was 2.53 W·kg^−1^ in older men (specificity = 78%; sensitivity = 62%; AUC = 0.730; *p* < 0.001) (Figure 2A) and 2.01 W·kg^−1^ in older women (specificity = 79%; sensitivity = 63%; AUC = 0.778; *p* < 0.001) (Figure 2B).

**FIGURE 3:**
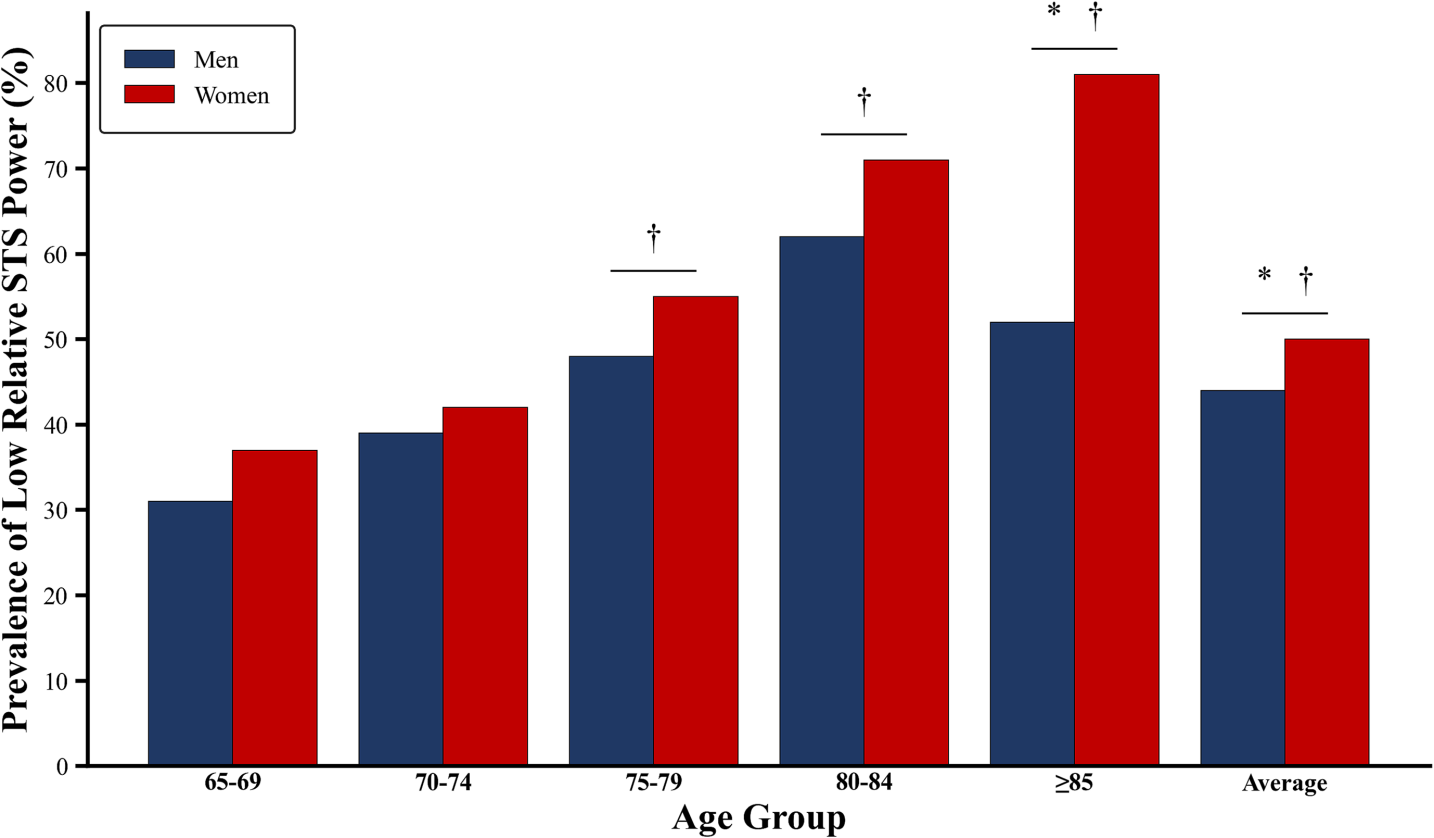
Prevalence of low relative STS power in older men and older women across different quinquennial age groups. *Note:* * Significantly different compared with men within the same age group (*p* < 0.05). † Significantly different compared with 65‐ to 70‐year‐old group within the same sex (*p* < 0.05).

The prevalence of low 30‐s relative STS power was significantly lower in older men compared with older women (43.5% vs. 50%, respectively; *p* = 0.005) (Figure 3). The prevalence of low 30‐s relative STS power of participants that were able to rise from the chair increased with increasing age, showing a higher prevalence in the group of men and women aged 85 or older (51.9% and 81.1%, respectively), 80–84 years (62.0% and 71.1%, respectively) and 75–79 years (48.0% and 55.2%, respectively) than in men and women aged 65–69 (30.5% and 36.6%, respectively) (all *p* < 0.05). No significant differences in the prevalence of low 30‐s relative STS power between men and women aged 70–74 (39.0% and 42.0%, respectively) years and those aged 65–70 years (*p* > 0.05).

### 3.3 Association Between Low Relative 30‐s STS Power and Adverse Health Conditions

**FIGURE 4:**
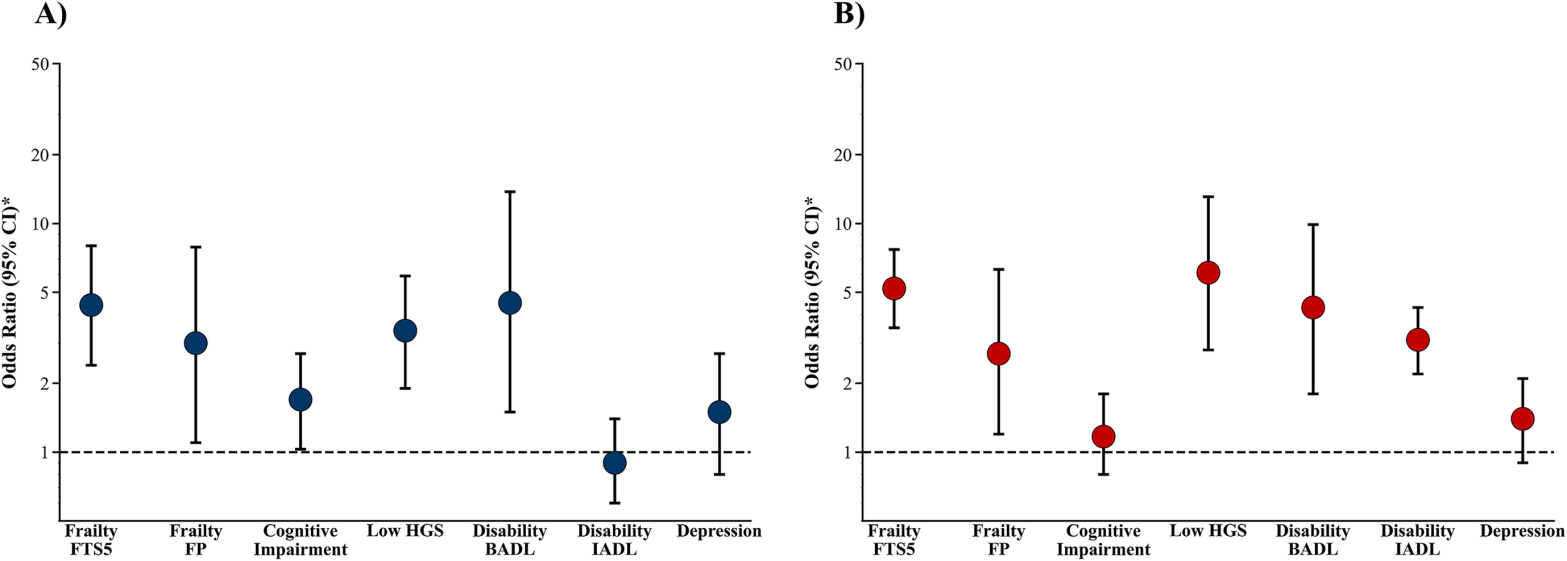
Logistic regression analysis showing the association between low relative STS power and adverse conditions in older men (A) and women (B). * Adjusted for age and comorbidities. BADL, basic activities of daily living; CI, confidence interval; FP, Fried frailty phenotype; FTS5, Frailty Trait Scale‐Short Form; HGS, habitual gait speed; IADL, instrumental activities of daily living.

The unadjusted analysis showed that low 30‐s relative STS power was associated with all the adverse conditions in both men and women except with depression (*p* = 0.101) and IADL (*p* = 0.629) in men (Table S1). After adjusting for age and comorbidities, low 30‐s relative STS power was associated with frailty assessed using both the FTS5 (OR [95% CI] = 4.4 [2.4–8.0]; *p* < 0.001) and FP (OR [95% CI] = 3.0 [1.1–7.9]; *p* = 0.026), cognitive impairment (OR [95% CI] = 1.7 [1.0–2.7]; *p* = 0.046), low HGS (OR [95% CI] = 3.4 [1.9–5.9]; *p* < 0.001) and disability in BADL (OR [95% CI] = 4.5 [1.5–13.8]; *p* = 0.009) in men (Figure 4A). No significant associations were found between low 30‐s relative STS power and depression (OR [95% CI] = 1.5 [0.8–2.7]; *p* = 0.190) or disability in IADL (OR [95% CI] = 0.9 [0.6–1.4]; *p* = 0.570) among older men. In older women, there was a significant association between low 30‐s relative STS power and frailty assessed using both the FTS5 (OR [95% CI] = 5.2 [3.5–7.7]; *p* < 0.001) and FP (OR [95% CI] = 2.7 [1.2–6.3]; *p* = 0.017), low HGS (OR [95% CI] = 6.1 [2.8–13.1]; *p* < 0.001), disability in BADL (OR [95% CI] = 4.3 [1.8–9.9]; *p* = 0.001) and IADL (OR [95% CI] = 3.1 [2.2–4.3]; *p* < 0.001) (Figure 4B). No significant associations were found between low 30‐s relative STS power and cognitive impairment (OR [95% CI] = 1.17 [0.8–1.8]; *p* = 0.463) or depression (OR [95% CI] = 1.4 [0.9–2.1]; *p* = 0.110) in women.

## 4 Discussion

The current study found that relative STS power, derived from the 30‐s STS test, differed significantly between sexes and decreased linearly with increasing age at a rate of −1.0% per year in men and −1.5% per year in women from the age of 65 onwards. Importantly, this is the first study to identify cut‐off points for low relative STS power using the 30‐s version of the STS test (2.53 W·kg^−1^ for men and 2.01 W·kg^−1^ for women) and to ascertain the prevalence of low 30‐s relative STS power among older men (43.5%) and women (50.0%) who were able to stand up from the chair. Moreover, older individuals with low 30‐s relative STS power showed higher odds of experiencing adverse conditions compared with those older adults with relative STS power values above the cut‐off points.

### 4.1 Decline in Relative 30‐s STS Power With Age

Cross‐sectional studies have indicated that unilateral leg power begins to decline earlier and at a faster rate than both muscle mass and muscle strength [S8, 26]. These studies showed that older people aged 85 or older exhibited ~44% less unilateral leg power than people aged over 65 years old, suggesting an average reduction of 2.2% per year in muscle power [26]. Recent longitudinal data revealed that both unilateral relative leg power and unilateral isometric leg strength begin to decline before the age of 30 years old [9]. Importantly, as indicated in both cross‐sectional [26] and longitudinal studies [9], unilateral leg power diminishes at a higher rate than unilateral leg strength, especially from the age of 65 onwards. In our study, we observed that 30‐s relative STS power in men and women aged > 85 years was on average, 25% lower than in the group of men and women aged 65–69 years, suggesting an average annual loss of −1.3%. The annual rate of decline observed in our study (older Spanish men and women) is slightly lower than that reported in studies conducted on other populations [27]. In this regard, studies on Danish populations have shown that the average annual rate of 30‐s relative STS power loss between 65 and 85 years in both men and women is −1.9% [27]. These differences between studies may be due to Danish older adults having, on average, higher levels of 30‐s relative STS power compared with Spanish older adults while exhibiting similar levels of 30‐s relative STS power in the lower percentiles [14], which could lead to higher annual rates of decline in the Danish population. Furthermore, the combination of a higher annual rate of 30‐s relative STS power loss and the broader age range (from 20 to > 85 years) evaluated contributed to the higher *R* ^2^ values observed in the study by Alcazar et al. [27] compared with ours. Importantly, this is the first study to demonstrate the prevalence of low relative STS power derived from the 30‐s STS test across different age groups. We observed an increase in the prevalence of older individuals with low 30‐s relative STS power with advancing age, rising from 31% in men and 37% in women aged 65–69 years to 52% in men and 81% in women aged > 85 years, respectively. In addition, this is the first study that reported the prevalence of low 30‐s relative STS power among older men and women across different age groups. The higher prevalence of low 30‐s relative STS power in women compared with men, particularly at advanced ages, may be attributed to the fact that women experienced a more pronounced decline in 30‐s relative STS power from the age of 80 onward in contrast to men who exhibited a deceleration in the loss of 30‐s relative STS power from 85 years of age. This deceleration was also noted in the study by Baltasar‐Fernandez et al. [15] and may be attributed to a survival effect, given that only 27 men were aged over 85 years in the current study.

### 4.2 Cut‐Off Points for Low Relative 30‐s STS Power Derived From the 30‐s STS Test

Several studies have reported cut‐off points for low 5‐rep relative STS power in recent years. In this regard, Alcazar et al. [14] demonstrated that the cut‐off points that best identified older individuals with mobility limitations were 2.6 W·kg^−1^ in men and 2.1 W·kg^−1^ in women. On the other hand, in the study by Baltasar‐Fernandez et al. [15], the cut‐off points that best identified older individuals with frailty were 2.5 W·kg^−1^ in men and 1.9 W·kg^−1^ in women, while in the study by Kirk et al. [16], the cut‐off points that best identified those older men and women experiencing recurrent falls and fractures were 2.0 and 1.6 W·kg^−1^, respectively. This variation in cut‐off points is primarily attributed to the specific outcome under consideration. It is reasonable that relative STS power cut‐off points are higher for identifying older individuals with mobility limitations than for identifying those with frailty and recurrent falls and fractures because the latter aspects represent a more advanced state of vulnerability. Reduced mobility has been shown to precede and exacerbate frailty [S9], and older individuals experiencing recurrent falls and fractures are typically in advanced stages of frailty [28]. It is important to note that all cut‐off points calculated in other investigations have been determined by implementing the 5‐repetition version of the STS test. Our study is the first to identify cut‐off points for low relative STS power using the 30‐s version of the STS test. In this context, 30‐s relative STS power cut‐off points that best identified older men and women with frailty were 2.53 and 2.01 W·kg^−1^, respectively. These values were highly similar to those reported by Baltasar‐Fernandez et al. [15] and Alcazar et al. [14] for identifying older individuals with frailty and mobility limitations using the 5‐rep STS test version, respectively. In addition, we should highlight the favourable AUC values yielded by 30‐s relative STS power in relation to its ability to identify older people with frailty (0.73 in men and 0.78 in women). This suggests that our cut‐off points can be implemented for the early detection of frailty in older individuals when the 30‐s STS test is used instead of the 5‐repetition STS test.

### 4.3 Low Relative 30‐s STS Power and Its Association With Adverse Conditions

Unilateral leg power has demonstrated a stronger association with physical function than muscle strength, additionally revealing that individuals with low unilateral leg power have double the risk of developing mobility limitations compared with those with low muscle strength [29]. Despite the significance of muscle power for older individuals, it remains a relatively neglected and unassessed parameter within clinical settings. This oversight may be attributed to the historical requirement for expensive instruments, necessitating data analysis or processing to quantify muscle power [30]. However, today, muscle power can be evaluated through the STS test (5‐repetition or 30‐s version) and the implementation of the Alcazar's equation [11, 19] and a smartphone app (Power Frail) [31], which has been validated against various instruments considered gold standards for muscle power assessment across diverse groups of older individuals (from well‐functioning to mobility‐limited) [12, 32]. Importantly, it has consistently proven to be the equation most strongly associated with adverse conditions among all equations available in the literature [13]. However, although the 5‐repetition STS test is commonly used, the 30‐s STS test, with its time‐based limit instead of a repetition‐based limit, may provide greater precision and allow for a more comprehensive assessment, especially in some populations. Specifically, the 30‐s STS test enables the evaluation of STS power in older adults with low physical function who may not be able to complete five STS repetitions in the 5‐rep STS test but can perform between one and four STS repetitions within the 30‐s STS version. This is particularly relevant, as between 2% and 26% of older people are unable to perform five STS repetitions [17]. However, although the 30‐s STS test may offer some advantages over the 5‐rep STS version, studies that have used the 5‐rep STS test have shown that older individuals with low 5‐rep relative STS power values exhibited an elevated risk of experiencing mobility limitations [14], frailty [15], disability in activities of daily living [33], reduced quality of life [15], hospitalizations [34] and even all‐cause mortality [34, 35]. The association reported in the literature between low 5‐rep relative STS power and various adverse conditions is corroborated in our study using the 30‐s version of the STS test, reinforcing the imperative of evaluating relative muscle power clinically through different versions of the STS test and the implementation of the Alcazar's equation [11]. It is important to note that this study is the first to demonstrate the association between low 30‐s relative STS power and cognitive impairment in older adults. In this sense, older men with low 30‐s relative STS power exhibited higher odds of experiencing cognitive impairment, whereas this association was not observed in women. These differences between older men and women may be attributed to the crucial role of the brain‐derived neurotrophic factor, which has been demonstrated to be a mediator between muscle and cognitive impairment, particularly in women [36, S10]. On the other hand, as observed in previous studies [S11, 37], the prevalence of depression was higher in women than in men in our study, which could explain the more pronounced decline in 30‐s relative STS power experienced by women. However, no association was found between low 30‐s relative STS power and depression in either women or men, suggesting the higher prevalence of depression in women may be influenced by other factors, such as marital status, anxiety or genetic factors [37, S12]. In addition, it is important to note that low 5‐rep relative STS power has been recently demonstrated to be a stronger predictor of adverse conditions than sarcopenia [10, 33]; however, the assessment of sarcopenia is much more integrated into clinical practice than the evaluation of relative muscle power. Moreover, STS power can be effectively used to monitor short‐ and long‐term changes following the implementation of exercise programmes, even in clinical settings [38, 39]. In this context, changes of 0.42 W·kg^−1^ in men and 0.33 W·kg^−1^ in women have been suggested as minimal clinically important changes [14]. Therefore, considering the high relevance of relative STS power for older individuals, we strongly recommend its use and inclusion in comprehensive geriatric assessment. By applying the identified cut‐off points for low 30‐s relative STS power and utilizing an operational algorithm [14], we will be able to detect specific deficits and recommend effective interventions to enhance relative STS power. This approach will facilitate the early identification of older individuals at risk of experiencing adverse conditions, thereby improving both their short‐term and long‐term prognosis.

## 5 Study Limitations

This study had some limitations that should be considered. First, the sample size of the oldest age groups was relatively small, which may be attributed to a survival effect. Then, the cut‐off points in this study were identified in a Spanish cohort, and thus, their applicability to the older population in other countries should be contrasted. However, it is noteworthy that the cut‐off points from our study closely resemble those reported in a cohort of 10 000 older individuals from various European countries [14]. Finally, given the cross‐sectional nature of this study, further investigations are required to understand the possible relationships and pathways between low relative STS power and the analysed adverse conditions and outcomes.

## 6 Conclusions

The present study revealed a decline in 30‐s relative STS power with age in both men and women. The reduction in 30‐s relative STS power was more pronounced in women than in men aged > 85 years, leading to a higher prevalence of low 30‐s relative STS power in women compared with men. The sex‐specific cut‐off points for low 30‐s relative STS power effectively discriminated those older adults with frailty and were significantly associated with adverse conditions in men and women. Therefore, relative STS power cut‐off points derived from the 30‐s STS test can be utilized to categorize and monitor older individuals throughout the ageing process.

## Ethics Statement

The authors certify that they comply with the ethical guidelines for authorship and publishing in the *Journal of Cachexia, Sarcopenia and Muscle* [40].

## Conflicts of Interest

The authors declare no conflicts of interest.

## Supporting information


**Data S1.** Supporting Information.

**Table S1.** Unadjusted and age‐adjusted associations of low relative STS power with adverse events.
