# Supplementary material for: Cut‐Off Points for Low Relative 30‐s Sit‐to‐Stand Power and Their Associations With Adverse Health Conditions
Source: J Cachexia Sarcopenia Muscle. 2025 Jan 10;16(1):e13676. doi: 10.1002/jcsm.13676 (PMC11718219; doi:10.1002/jcsm.13676)
Supplement: Supplementary file 1 — Data S1. Supporting Information. [file JCSM-16-e13676-s001.docx]

**Supplementary references:**

**S1. Woolf SH, Schoomaker H. Life Expectancy and Mortality Rates in the United States, 1959-2017. JAMA. 2019;322:1996-2016.**

**S2. García-García FJ, Carcaillon L, Fernandez-Tresguerres J, Alfaro A, Larrion JL, Castillo C, et al. A new operational definition of frailty: the Frailty Trait Scale. J Am Med Dir Assoc. 2014;15:371.e7-.e13.**

**S3. Rockwood K, Mitnitski A. Frailty in relation to the accumulation of deficits. J Gerontol A Biol Sci Med Sci. 2007;62:722-7.**

**S4. Vermeiren S, Vella-Azzopardi R, Beckwée D, Habbig AK, Scafoglieri A, Jansen B, et al. Frailty and the Prediction of Negative Health Outcomes: A Meta-Analysis. J Am Med Dir Assoc. 2016;17:1163.e1-.e17.**

**S5. Shafiee G, Keshtkar A, Soltani A, Ahadi Z, Larijani B, Heshmat R. Prevalence of sarcopenia in the world: a systematic review and meta- analysis of general population studies. J Diabetes Metab Disord. 2017;16:21.**

**S6. Jones CJ, Rikli RE, Beam WC. A 30-s chair-stand test as a measure of lower body strength in community-residing older adults. Res Q Exerc Sport. 1999;70:113-9.**

**S7. Shin C, Park MH, Lee SH, Ko YH, Kim YK, Han KM, et al. Usefulness of the 15-item geriatric depression scale (GDS-15) for classifying minor and major depressive disorders among community-dwelling elders. J Affect Disord. 2019;259:370-5.**

**S8. Lauretani F, Russo CR, Bandinelli S, Bartali B, Cavazzini C, Di Iorio A, et al. Age-associated changes in skeletal muscles and their effect on mobility: an operational diagnosis of sarcopenia. J Appl Physiol (1985). 2003;95:1851-60.**

**S9. Eeles E, Low Choy N. Frailty and Mobility. Interdiscip Top Gerontol Geriatr. 2015;41:107-20.**

**S10. Delezie J, Weihrauch M, Maier G, Tejero R, Ham DJ, Gill JF, et al. BDNF is a mediator of glycolytic fiber-type specification in mouse skeletal muscle. Proc Natl Acad Sci U S A. 2019;116:16111-20.**

**S11. Rodda J, Walker Z, Carter J. Depression in older adults. BMJ. 2011;343:d5219.**

**S12. Hung YC, Chen YH, Lee MC, Yeh CJ. Effect of Spousal Loss on Depression in Older Adults: Impacts of Time Passing, Living Arrangement, and Spouse's Health Status before Death. Int J Environ Res Public Health. 2021;18.**
