# Supplementary material for: Cut‐Off Points for Low Relative 30‐s Sit‐to‐Stand Power and Their Associations With Adverse Health Conditions
Source: J Cachexia Sarcopenia Muscle. 2025 Jan 10;16(1):e13676. doi: 10.1002/jcsm.13676 (PMC11718219; doi:10.1002/jcsm.13676)
Supplement: Supplementary file 2 — Table S1. Unadjusted and age‐adjusted associations of low relative STS power with adverse events. [file JCSM-16-e13676-s002.docx]

**Supplementary Table S1.** Unadjusted and age-adjusted associations of low relative STS power with adverse events

|  | **Men** | | | **Women** | | |
| --- | --- | --- | --- | --- | --- | --- |
|  | **OR** | **95 % CI** | ***p* value** | **OR** | **95 % CI** | ***p* value** |
| **Unadjusted model** |  |  |  |  |  |  |
| **Frailty FTS5** | 5.530 | 3.240 – 9.739 | <0.001 | 6.371 | 4.426 – 9.170 | <0.001 |
| **Frailty FP** | 4.838 | 1.926 – 12.16 | 0.001 | 4.549 | 2.073 – 9.982 | <0.001 |
| **Cognitive Impairment** | 1.884 | 1.194 – 2.973 | 0.006 | 1.719 | 1.199 – 2.466 | 0.003 |
| **Depression** | 1.613 | 0.911 – 2.856 | 0.101 | 1.649 | 1.144 – 2.377 | 0.007 |
| **Disability in BADL** | 6.200 | 2.075 – 18.53 | 0.001 | 5.992 | 2.898 – 12.390 | <0.001 |
| **Disability in IADL** | 1.105 | 0.738 – 1.653 | 0.629 | 3.747 | 2.773 – 5.064 | <0.001 |
| **Low HGS** | 4.227 | 2.472 – 7.227 | <0.001 | 9.834 | 4.656 – 20.77 | <0.001 |
| **Adjusted by age** |  |  |  |  |  |  |
| **Frailty FTS5** | 4.727 | 2.646 – 8.445 | <0.001 | 5.204 | 3.573 – 7.579 | <0.001 |
| **Frailty FP** | 3.885 | 1.524 – 9.900 | 0.004 | 3.298 | 1.471 – 7.397 | 0.004 |
| **Cognitive Impairment** | 1.639 | 1.027 – 2.616 | 0.038 | 1.314 | 0.893 – 1.923 | 0.160 |
| **Depression** | 1.558 | 0.874 – 2.779 | 0.133 | 1.489 | 1,020 – 2.173 | 0.039 |
| **Disability in BADL** | 5.235 | 1.737 – 15.78 | 0.003 | 4.008 | 1.897 – 8.467 | <0.001 |
| **Disability in IADL** | 0.984 | 0.651 – 1.488 | 0.941 | 2.931 | 2.130 – 4.032 | <0.001 |
| **Low HGS** | 3.794 | 2.206 – 6.525 | 0.000 | 7.860 | 3.693 – 16.73 | <0.001 |
| OR: odd ratio; CI: confidence interval; FTS5: Frailty Trait Scale 5 ≥ 25 points; FP: Frailty Phenotype ≥ 3 criteria; BADL: basic activities of daily living; IADL: instrumental activities of daily living; HGS: habitual gait speed. | | | | | | |
